# A comparative study of the use of digital technology in the anterior smile experience

**DOI:** 10.1186/s12903-024-04228-3

**Published:** 2024-04-25

**Authors:** Jiayi Liu, Maihepireti Maihemaiti, Lijuan Ren, Mierzhati Maimaiti, Nan Yang, Yuan Wang, Minxiang Wang, Xiaoping Wang, Yating Fu, Zhenhua Wang

**Affiliations:** 1 Prosthodontics Department of Urumqi Stomatological Hospital, Urumqi, 830002 Xinjiang China; 2 Urumqi Stomatological Hospital, Urumqi, 830002 Xinjiang China; 3 Xinjiang Key Laboratory of Traditional Chinese Medicine Ethnic Medicine, Pharmaceutical Common Key Technology Research, Urumqi, 830011 Xinjiang China

**Keywords:** 3D printing, Crown lengthening, Digital smile design (DSD), Gingivoplasty, Digital dual positioning guides

## Abstract

**Objectives:**

this study aims to compare the clinical outcomes of traditional and digital crown extension guides in the aesthetic restoration of anterior teeth. Additionally, the study will analyze the differences in the results of various digital crown extension guides in anterior aesthetic restorations.

**Methods:**

Sixty-two patients who required aesthetic restoration of their anterior teeth were selected for this study. The patients had a total of 230 anterior teeth and were randomly divided into three groups: a control group of 22 cases who received diagnostic wax-up with pressure film, an experimental group 1 of 20 cases who received 3D printed digital models with pressure film, and an experimental group 2 of 20 patients who received digital dual-positioning guides. The control group had a total of 84 anterior teeth, experimental group 1 had 72 anterior teeth, and experimental group 2 had 74 anterior teeth. The study compared three methods for fabricating crown extension guides: the control group used the diagnostic wax-up plus compression film method, while experimental group 1 used compression film on 3D printed models and experimental group 2 used 3D printed digital dual-positioning crown extension guides. After the crown lengthening surgery, the control group patients wore DMG resin temporary crown material for gingival contouring, while the experimental group patients wore 3D printed resin temporary crowns for the same purpose. The patients were followed up in the outpatient clinic after wearing temporary crowns for 1 month, 3 months, and 6 months, respectively. The clinical results were evaluated in terms of marginal fit, red aesthetic index, and white aesthetic index.

**Results:**

Based on the statistical analysis, the experimental group required significantly fewer follow-up visits and less time for guide design and fabrication compared to the control group. Additionally, the surgical time for the experimental group was significantly shorter than that of the control group. During the postoperative period between the 1st and 3rd month, the PES index scores for the marginal gingival level, proximal, and distal mesiodistal gingival papillae of the experimental group showed a trend of superiority over those of the control group. By the 6th month, the marginal gingival level exhibited a significant difference between the experimental and control groups. The experimental group demonstrated superior results to the control group in terms of shape, contour, and volume of the teeth, color, surface texture, and transparency of the restorations, and features during the 1st and 3rd postoperative months. In the 6th month, the comparative results indicated that the experimental group continued to exhibit superior outcomes to the control group in terms of the shape, color, surface texture, and transparency of the restorations, as well as the characteristics of the teeth. Additionally, the experimental group demonstrated significantly fewer gingival alterations than the control group at 1 month, 3 months, and 6 months post-procedure, with this difference being statistically significant. Furthermore, the combination of 3D printing technology and restorative techniques was utilized, resulting in consistent patient satisfaction.

**Conclusion:**

Digitalisation plays an important role in anterior aesthetic restorations. The use of digital technology to manage the entire process of anterior cosmetic restorations can improve restorative results, reduce the number of follow-up appointments, shorten consultation time, and achieve better patient satisfaction.

## Introduction

The aesthetics of the front teeth is no longer limited to focusing on the color, shape, and alignment of the teeth, but includes the art of improving the relationship between the teeth and gums and the alignment of the teeth to express a more perfect smile. The smile has become an important part of the aesthetic appearance and is an important sign of beauty and health. Many factors determine the overall aesthetics of a smile. In the context of anterior aesthetics, there is a group of patients who present with a gingival smile, passive under-eruption, clinical crowns that are too short, gingival margin asymmetry, periodontal inflammation due to the destruction of the biological width by poor restorations, and other conditions that may be present to varying degrees, and other conditions that affect the smile to varying degrees and require modification of the gingival margins and alveolar bone position by periodontal surgery or other methods, as well as addressing esthetic conditions in the anterior region to achieve aesthetic and health-conscious stabilization. Hard and soft tissues. In these complex anterior esthetic cases, the surgeon must perform an esthetic design and facial analysis of the patient, as well as a risk assessment, including the position of the midline, the height of the mouth and lips, the symmetry of the gingival margins, the height of the smile line, and the shape and color of the teeth. Within these aesthetic design options, crown lengthening is often used clinically to improve the aesthetics of the anterior teeth. With the continuous improvement of living standards, doctors and patients are seeking an efficient, comfortable, precise as well as minimally invasive surgical method to achieve a long-lasting and stable surgical effect; therefore, digital medicine was born, and through the application of digital technology design guidance, it can help doctors to achieve a more stable and precise aesthetic restoration effect in the clinic [1–3].

Through clinical research, the group intends to produce different 3D printed crown extension surgical guides and post-operative temporary restorations, and through clinical research, compare the effects of aesthetic restoration of anterior teeth using digital and traditional restorative techniques, to provide a more theoretical basis for personalized aesthetic restorations of the 'red and white' oral cavity.

## Information and methods

### General information

Sixty-two patients requiring aesthetic restoration of anterior teeth with a total of 230 anterior teeth received between January 2022 and June 2023 were selected and randomly divided into 22 cases with a total of 84 anterior teeth in the traditional crown extension guide group (control group), 20 cases with a total of 72 anterior teeth in the 3D-printed digitally modeled pressure film group (experimental group 1), and 20 patients with a total of 74 anterior teeth in the digitally dual-positioned guide group (experimental group 2). General information such as age, gender, and tooth position(from the upper right to the upper left tooth) of the patients in the two groups were compared and the difference was not statistically significant (*P* > 0.05). See Table 1.

**Table 1:** Comparison of general information of the three groups

**General information**		**Control group**	**Eperimental group 1**	**Eperimental group 2**	***P***
Age		35.27 ± 8.87	34.09 ± 5.48	35.05 ± 5.60	0.840
Sex	male	8 (36.4%)	4 (19.0%)	6 (30.0%)	0.448
	female	14 (63.6%)	17 (81.0%)	14 (70.0%)	
Tooth position	11–21	6 (27.3%)	7 (33.3%)	6 (30.0%)	0.839
	12–22	11 (50.0%)	12 (57.1%)	11 (55.0%)	
	13–23	5 (22.7%)	2 (9.5%)	3 (15.0%)	

### Inclusion and exclusion criteria [4, 5]

Inclusion criteria: ① upper and lower anterior aesthetic zone, normal occlusion, no crowding, no malocclusion, no missing, normal tooth alignment; ② healthy periodontal tissue, no gingival recession, periodontal pockets PD ≤ 3 mm, bleeding index BI ≤ 2 (Mazza Bleeding Index, 1981); ③ crown fracture, oversized teeth, malocclusion, and other teeth with abnormal crown morphology; ④ presence of scattered gaps or slightly twisted teeth with unusual alignment; ⑤ Those requiring esthetic restorations for an open gum smile and incongruous crown/root ratio of the anterior teeth.⑥ Periodontal procedures such as guided tissue regeneration, root surface coverage, crown lengthening, soft tissue grafting, etc. have not been performed in the anterior region.

Exclusion criteria: ① gingivitis, periodontitis has not been controlled; ② trauma or carious teeth, the section is located in the subgingival greater than 3mm or root is shorter than 10mm; ③ occlusal disorders, patients with nocturnal grinding; ④ history of mental illness and in the episodes, can not cooperate with the person.

### Selection of research subjects

It has been shown [6–10] that there is a significant relationship between soft tissue morphology and age, and in this study, to minimize the effect of age on soft tissues, young and middle-aged people aged 18–50 years were selected, and to exclude the influence of gingival biotype on the results, patients with medium-thickness gingival phenotypes were included as subjects in all studies.

### Materials and instruments

Canon DSLR camera (EOS60D, Canon Japan), DSD software (Smilefy Inc., USA); 3shape Trios intraoral scanner; BEGO 3D printer (Germany); Liquid photosensitive resin (Varseowax) for print guide material; CBCT Cone-Beam CT (Kavo Kawa, Germany); Luxatemp provisional crown and bridge repair material (DMG, Germany);

### Research methods

**Fig. 1:**
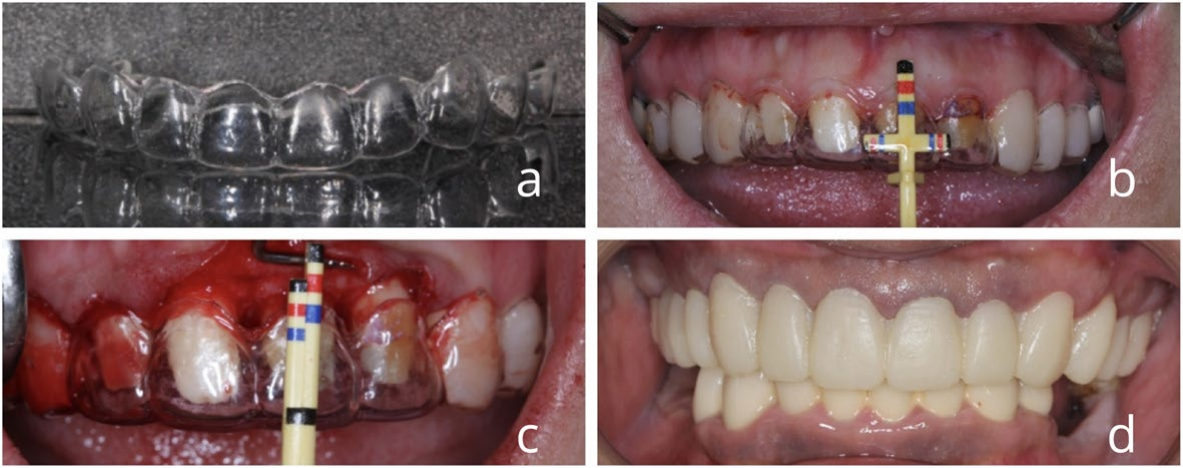
Diagnostic wax-up die guide plat (**a**) Handmade die guide plat (**b**) Surgical positioning of the die guide plat combined with the "shu" double-armed ruler (**c**) Crown lengthening surgery with the die guide plat combined with the "shu" double-armed ruler (**d**) Gingival contouring with a temporary crown made of DMG resin

**Fig. 2:**
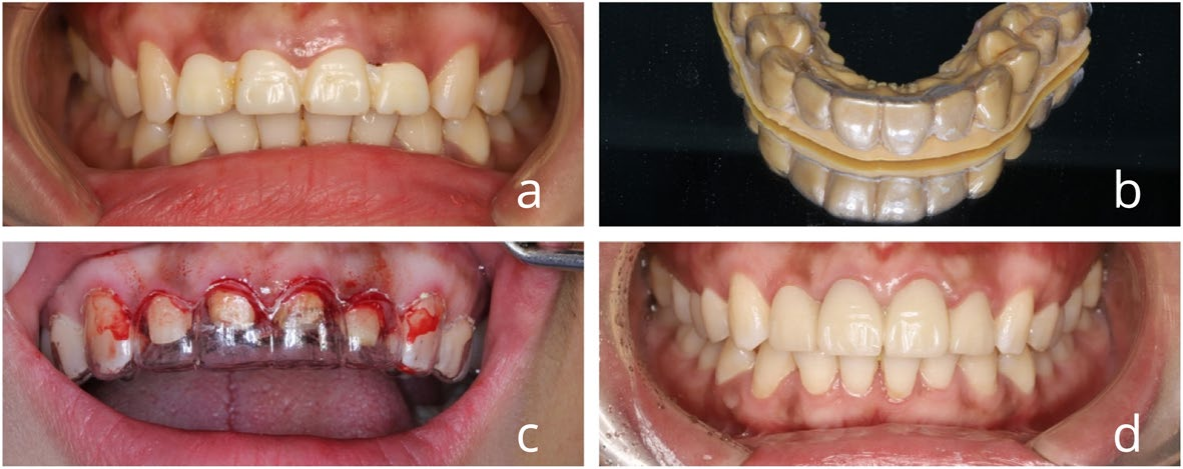
Clinical flowchart of digital compression membrane guide. **a** Preoperative intraoral photograph (**b**) 3D printed model after digital design, pressure film fabrication of crown lengthening guide (**c**) Crown lengthening surgery guided by digital pressure film guide (**c**) Gingival contouring by wearing a 3D printed temporary crown

**Fig. 3:**
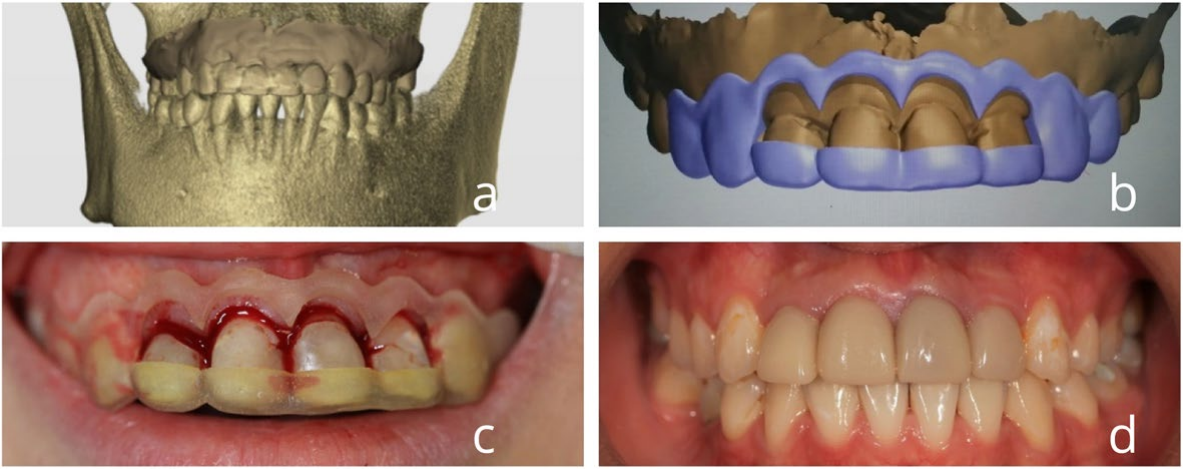
Dual positioning guide design and performing surgery. **a**-**b** Fitting CBCT and oral scan data and design (**c**) Crown lengthening surgery guided by digital dual positioning guides (**c**) Gingival contouring by wearing a 3D printed temporary crown

The experiment was divided into 3 groups, control group: traditional compression foil guides, and DMG resin temporary crowns (Fig. 1); the experimental group was divided into 2 groups, experiment 1 (Fig. 2): compression crown extension guides and 3D printed temporary crowns using digitally designed 3D printed models. Experimental group 2 (Fig. 3): 3D printed crown extension double positioning guides and 3D printed temporary crowns.

### Control group

- (1) Pre-operative alginate is taken from the conventional model and photographs of the patient are taken inside and outside the mouth.
- (2) The surgeon designs a diagnostic wax-up and makes a pressure film guide on the diagnostic wax-up.

The periodontist performs the periodontal crown lengthening surgery under the guidance of the pressure film guide: The incision in the affected area is positioned according to the ideal gingival margin position on the guide and the excess gingiva is excised; the guide is removed, the flap is incised and the alveolar bone is exposed, and the position of the bone apex in the operation requires a combination of the indication of the periodontal probe [11, 12] and a combination of the use of the shu two-armed ruler to indicate the position of the bone crest apex [13], and the alveolar bone is removed using a bone chisel/bullet drill so that the gingival margin is at least 3 mm from the crest of the alveolar ridge to match the biological width and is displaced towards the crest of the adjacent teeth.

- (4) Fabricate the temporary restoration: Remove the sutures after 1 week and refer to a prosthodontist for tooth preparation and chairside fabrication of DMG resin temporaries concerning the diagnostic wax-up.
- (5) Follow-up observation: Wear the temporary crown after surgery and record the amount of gingival change, red and white aesthetic index and patient satisfaction at 4 weeks, 3 months and 6 months to evaluate the clinical restoration effect. Since the gingiva will change over time after crown extension surgery, the prosthodontist should adjust the shape of the temporary crown according to the patient's intraoral situation when the patient wears it for inspection, so that the gingiva is better guided and shaped.

### Digital design group (experimental group)

#### Experimental group 1

1. First intraoral scan

The first scan of the patient's intraoral condition is performed by the patient's first prosthodontist using the 3shape Trios intraoral scanner to obtain an electronic model;

(2) Aesthetic design

The scanned digital model is imported into the restorative software (3shape Dental system), DSD aesthetic design, and the aesthetic functional gingival morphology is designed;

(3) Referring to the DSD aesthetic design using design software in the patient's mouth scanned model into the virtual crown, to determine the boundaries of the anterior gingival resection, by the principle of medium-thickness gingival resection in the gingiva under the 3 mm [14], the virtual crown edge to the gingiva to extend 3 mm, to determine the resection boundary of the alveolar bone;
(4) The design was completed to output STL format to 3-shape CAM bridge typesetting software;
(5) The typeset data were imported into a German BEGO 3D printer (Varseo 3D printing system), 3D models were printed using liquid photosensitive resin, and crown extension guides (one gingival guide and one osteotomy guide) were pressed on the 3D models;
(6) The periodontist performed the crown lengthening procedure according to the incisal gingival and alveolar bone guides;
(7) After removing the wires and transferring them to the prosthodontist to prepare the teeth, scan the prepared model again, import the prosthodontist software (3shape dental system), and design the shape of the temporary restoration;
(8) Design completed output STL format to 3shape CAM bridge typesetting software, typesetting imported into the German BEGO 3D printer, the use of liquid sensitized resin material to print temporary restorations;
(9) 3D-printed temporary restorations were brought into the patient's mouth, and the number of gingival changes and clinical restorative effects were observed at 4 weeks, 3 months, and 6 months after surgery(see Fig. 2).

#### Experimental group 2

1. Digital data acquisition: Intraoral photos of the patient's dentition (frontal and 45° and 90° occlusal photos) and extraoral photos (frontal resting jaw, frontal, 45°, and 90° smile photos) were taken using a DSLR camera. The alveolar bone morphology was obtained using CBCT, and the digital information of the dentition and gingiva in the oral cavity was obtained using an oral scanner.
2. Aesthetically designed crowns with extended dual positioning guides: The restorative software (3shape Dental system) imports the patient's facial photographs and scanned digital models, considering the aesthetic aspect ratio of the anterior teeth, overall dental characteristics, and patient preferences. The software uses the Digital Smile Design (DSD) to create a personalized design for tooth shape, color, and gingival morphology. The facial smile photographs are used to generate a 3D smile effect diagram, which is then presented to the patient for confirmation or modification before proceeding to the next step. The target tooth model is fitted with CBCT (Fig. 1), and the bone tissue morphology is designed according to the principle of periodontal biological width.
3. 3D printing of crown extension dual guides: The design was output in STL format and imported into the German BEGO 3D printer (Varseo 3D printing system). The liquid photosensitive resin (Varseowax) was used to print the crown extension dual-positioning guides, which guide the resection of the gingiva and alveolar bone.
4. The periodontal crown lengthening surgery was performed by the periodontist with the assistance of the dual positioning guide.
5. Design of 3D printed temporary restorations: Once the stitches have been removed, the dentist will transfer the design to the prosthodontist for dental preparation. The oral cavity will then be scanned again to obtain the dental preparation model, which will be imported into the prosthodontist's design software (3Shape Dental System). The shape of the temporary restorations will be designed according to the color of the target crowns and gingival morphology. The design will then be output in STL format to the 3Shape CAM bridge software and imported into the German BE BE. After completing the design, it was imported into the BEGO 3D printer in Germany, and the temporary restoration was printed using liquid-sensitized resin (see Fig. 3).

The patients wore the 3D-printed temporary restorations for gingival contouring and the temporary crowns for 1, 3, and 6 months after the operation to record the amount of gingival changes and clinical restorative effects.

Risk and variability control, all periodontal surgeries in this project were performed by the same periodontist and tooth preparation was performed by the same prosthodontist.

#### Indicators of clinical detection

1. The study recorded the clinical operation time, number of follow-up visits, and design and fabrication time of guides and temporary crowns in three patient groups. The occurrence of second-hand surgery was compared and studied among the groups.
2. A gingival margin stability test was conducted by calculating the amount of change in gingival margin position at the mean site from the difference in crown heights at 1, 3, and 6 months after crown lengthening.
3. PES [15] red aesthetic score (pinkesthetcscore): This score evaluates the following aspects of the gingival tissue: ① proximal mesial gingival papilla, ② distal mesial gingival papilla, ③ marginal gingival level, ④ soft tissue morphology, ⑤ alveolar eminence shape, ⑥ soft tissue color, and ⑦ soft tissue texture. Each index is rated on a scale of 0 to 2, with 2 indicating good evaluation parameters, 1 indicating moderate, and 0 indicating poor.
4. The White Esthetic Score (WES) [15] is scored based on five aspects: crown morphology, crown profile, crown color, crown surface texture, and transparency/personalization. Each indicator is given a score of 2 for no difference, 1 for a small difference, and 0 for a large difference with neighboring teeth.
5. The VAS [16], or visual analog scale, allows patients to evaluate the peripheral soft tissue morphology curvature, the color texture of the restoration, crown morphology, color, overall aesthetic effect, and the cost of the number of visits. The score ranges from 1 to 100, with higher scores indicating greater satisfaction.

### Statistical analysis

SPSS version 26.0 and GraphPad Prism software were utilized for data collection and statistical analysis in this study. The measurement data were expressed as mean ± standard deviation $\left(\overline{x}\pm \mathrm{s }\right)$ if they followed a normal distribution. For data that did not follow a normal distribution, the mean ± standard deviation was used, and ANOVA analysis was performed for group comparisons. For data that did not follow a uniform variance, Dunnett's T3 multiple comparisons were used for group comparisons. The rates or component ratios of count data were analyzed using chi-square tests. The rank-sum test was used to analyze ordered multicategorical data. A statistically significant difference was considered when *P* < 0.05.

### Typical case

**Fig. 4:**
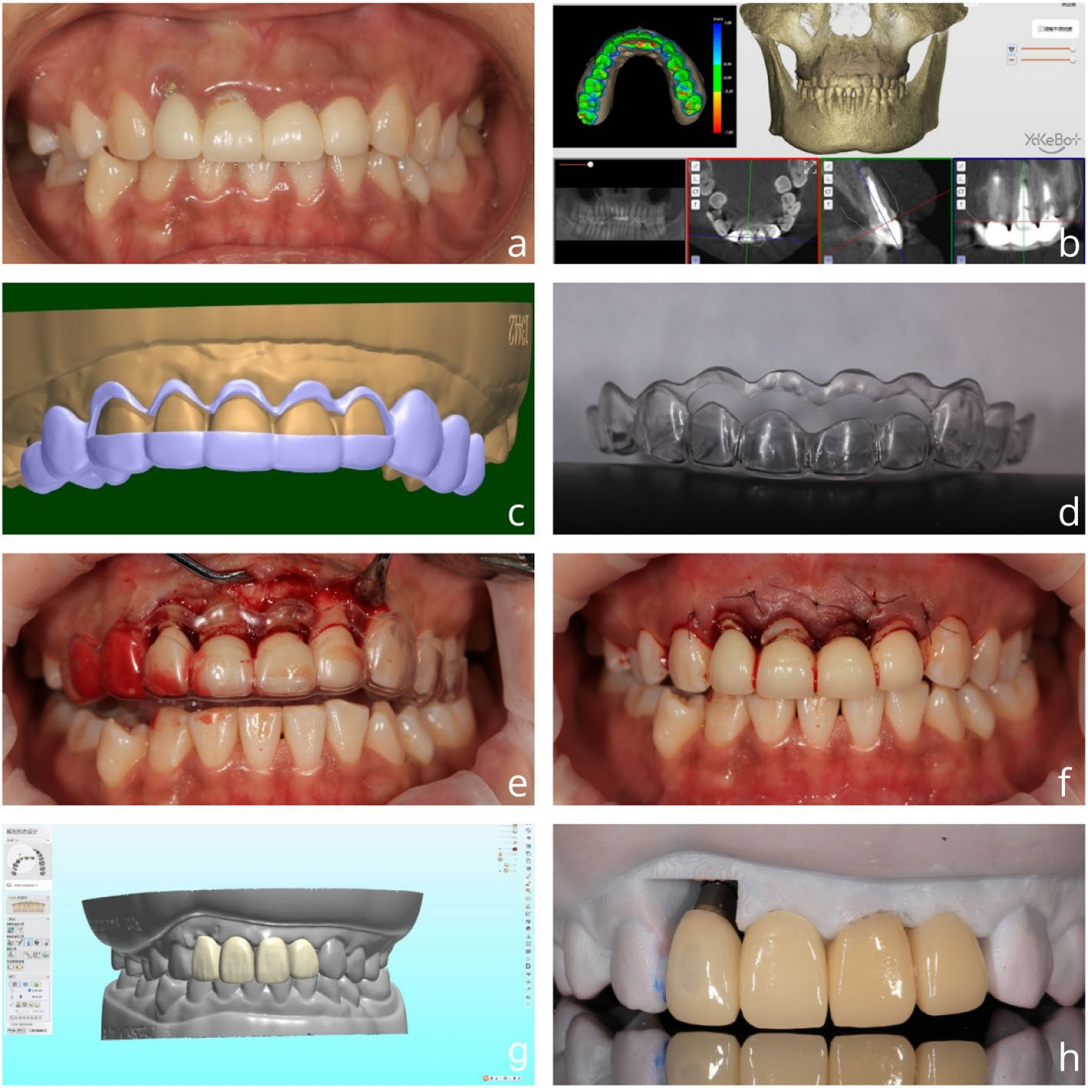
**a** Preoperative (**b**-**c**) digital design process (**d**) Dual positioning of the crown lengthening guide (**e**–**f**) Crown lengthening procedure guided by the guide (**g**) Design of the provisional crown (**h**) 3D printing of the provisional crown

**Fig. 5:**
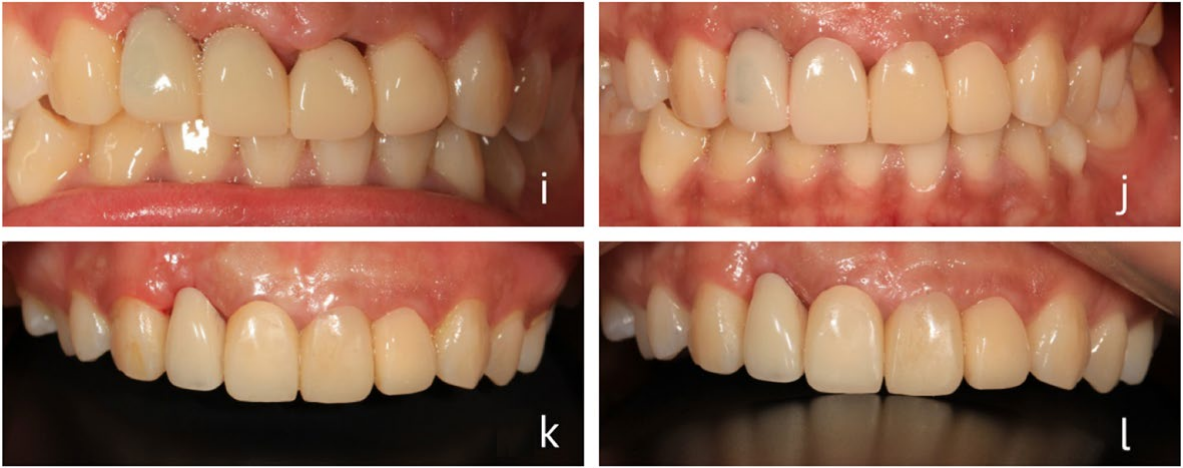
Shaping process of wearing 3D printed temporary crowns (**i**) Immediately after surgery (**j**) Wearing temporary crowns for 4 weeks (**k**) Wearing temporary crowns for 3 months (**l**) Wearing temporary crowns for 6 months

Guo, a 32-year-old female employee, presented at our hospital due to an unsightly porcelain bridge on her anterior teeth. Upon oral examination, we found no facial deformities, defects, swelling, or fistulas, and no numbness upon touch. Bilateral movement of the temporomandibular joints was symmetrical, with no pressure pain, popping, or limitation of mouth opening. Intraoral examination revealed… The patient presents with fixed restorations on teeth 11, 12, 21, and 22, with crown margin incompatibility. Additionally, there is labial crown margin erythema on teeth 21 and 22, and on teeth 11 and 12 with pus overflow. Imaging reveals a discontinuous high-density image in the root canal of tooth 12, and a low-density image visible at the root tip. The diagnosis includes tooth defects on teeth 1, 11, 12, 21, and 22, marginal gingivitis on teeth 12–22, and apical periodontitis on tooth 12. The treatment plan is to be determined. The proposed treatment plan includes 1) Referral to periodontics for systemic periodontal treatment; 2) Aesthetic DSD design of anterior teeth and creation of a dual-positioning crown lengthening guide for the patient; 3) Crown lengthening surgery guided by the aforementioned guide; 4) Referral to the internal medicine department for further treatment of tooth 12; 5) Wearing 3D printed temporary crowns for 6 months; 6) Placement of all-porcelain restorations for final restoration. It should be noted that during the crown lengthening surgical flap procedure, an occult fracture of the root surface of tooth 12 was discovered. As a result, it was recommended that tooth 12 be extracted and restored with an implant at a later date. Please refer to Figs. 4 and 5 for details of the treatment process.

## Result

### Differences between the three groups of patients in terms of clinical procedure time, number of follow-up appointments, and time to design and fabricate guides and temporary crowns

The statistical analysis revealed a significant difference (*P* < 0.01) between patients in the experimental group 1 and the control group regarding the number of revisits and the guide plate design and fabrication time. However, there was no significant difference (*P* > 0.05) in terms of the duration of surgery. Furthermore, a statistically significant difference (*P* < 0.01) was observed between patients in experimental group 2 and the control group in terms of operation time, number of follow-up visits, guide plate design and fabrication time see Table 2.

**Table 2:** shows the statistical results of the control and experimental groups in terms of operation time, number of follow-ups, guide plate design, and fabrication time

**Clinical indicators**	**Control group**	**Eperimental group 1**	**Eperimental group 2**
Operation time	67.27 ± 11.62	59.52 ± 9.73	45.00 ± 7.61^**^
Number of follow-up visits	8.91 ± 1.72	6.09 ± 0.94^**^	6.15 ± 0.49^**^
Guide plate design and fabrication time	10.96 ± 1.56	2.71 ± 0.56^**^	4.70 ± 0.73^**^

Compared to control group

^*^P < 0.05

^**^P < 0.01

### Comparison of PES index score

**Fig. 6:**
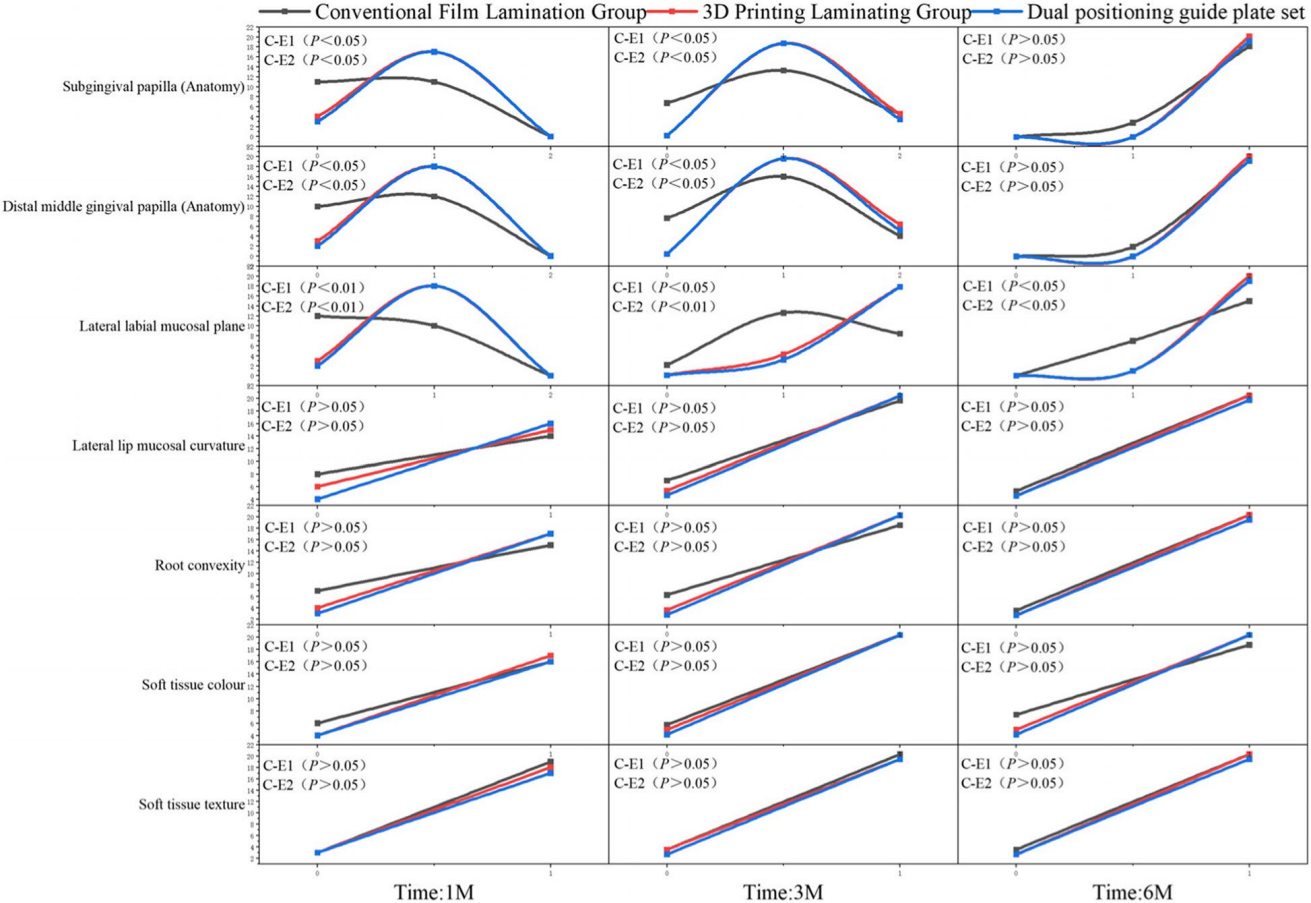
Results of the PES index score.C:control group;E1:experimental group1;E2:experimental group2

At 4 weeks and 3 months after the operation, there was a statistically significant difference in the PES index scores of the marginal gingival level, proximal mesial gingival papilla, and distal mesial gingival papilla between the 3D-printed compression membrane group and dual-positioning guide group compared to the conventional compression membrane group (*P* < 0.05, *P* < 0.05, *P* < 0.01, respectively). At 6 months, the experimental group showed a statistically significant improvement in marginal gingival level compared to the control group (*P* < 0.05). However, there were no statistically significant differences in soft tissue colour, labial mucosal curvature, root protrusion, and soft tissue texture between the two groups (*P* > 0.05), as shown in Fig. 6.

### Comparison of WES index score

**Fig. 7:**
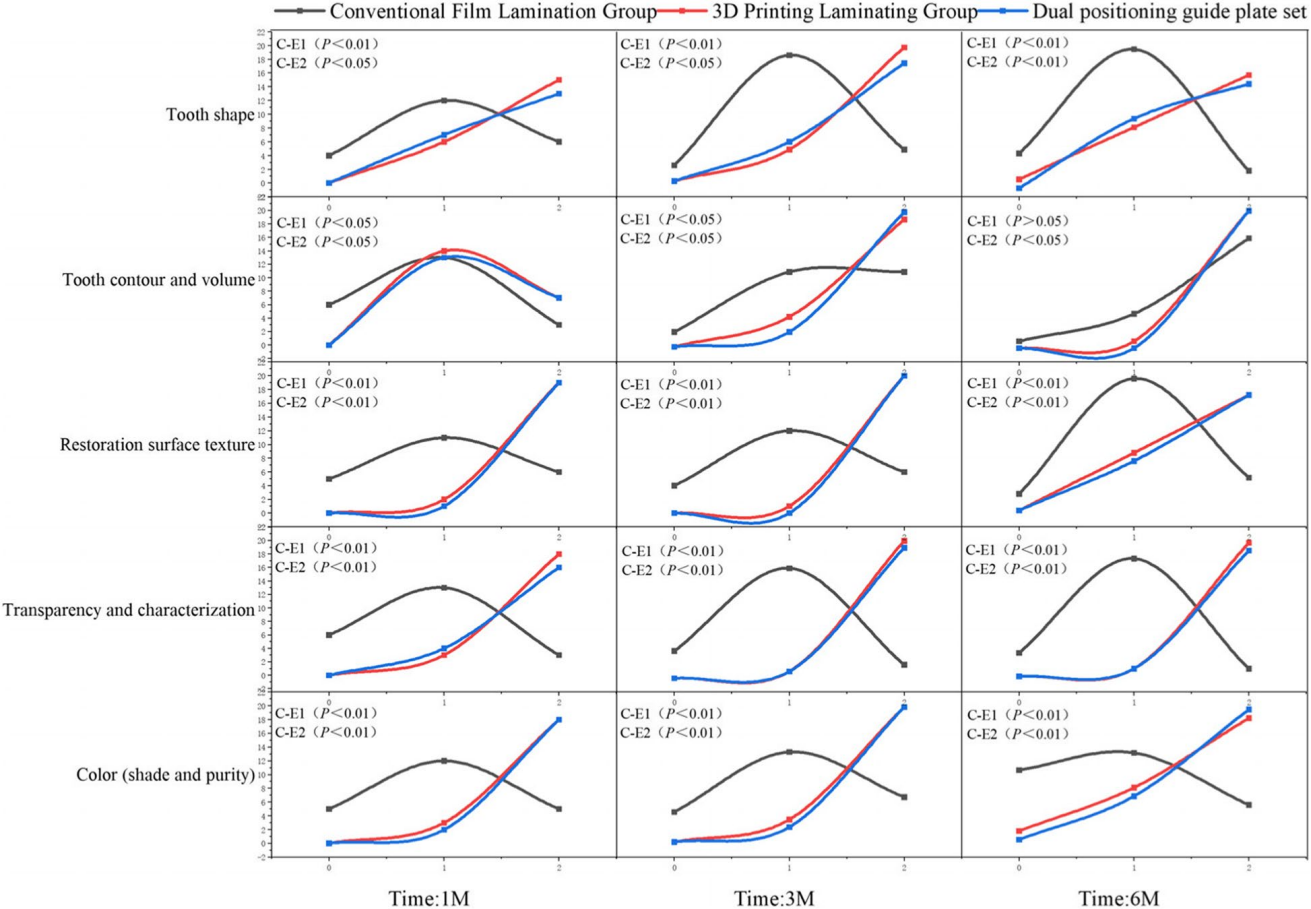
Results of WES index score.C:control group;E1:experimental group1;E2:experimental group2

At postoperative months 1 and 3, statistically significant differences were observed in the WES index scores for tooth shape, tooth contour, volume, color (hue and purity), restoration surface texture and transparency, and character between the 3D printed laminate group and the dual-positioning guide group when compared to the conventional laminate group (*P* < 0.05 and *P* < 0.01, respectively). At month 6, the comparison of tooth contour and volume did not show a statistically significant difference (*P* > 0.05). However, the comparison of tooth shape, color (shade and purity), restoration surface texture, transparency, and features showed statistically significant differences (*P* < 0.01). Specific data can be found in Fig. 7.

### Comparison of the amount of gingival changes in the three groups

**Fig. 8:**
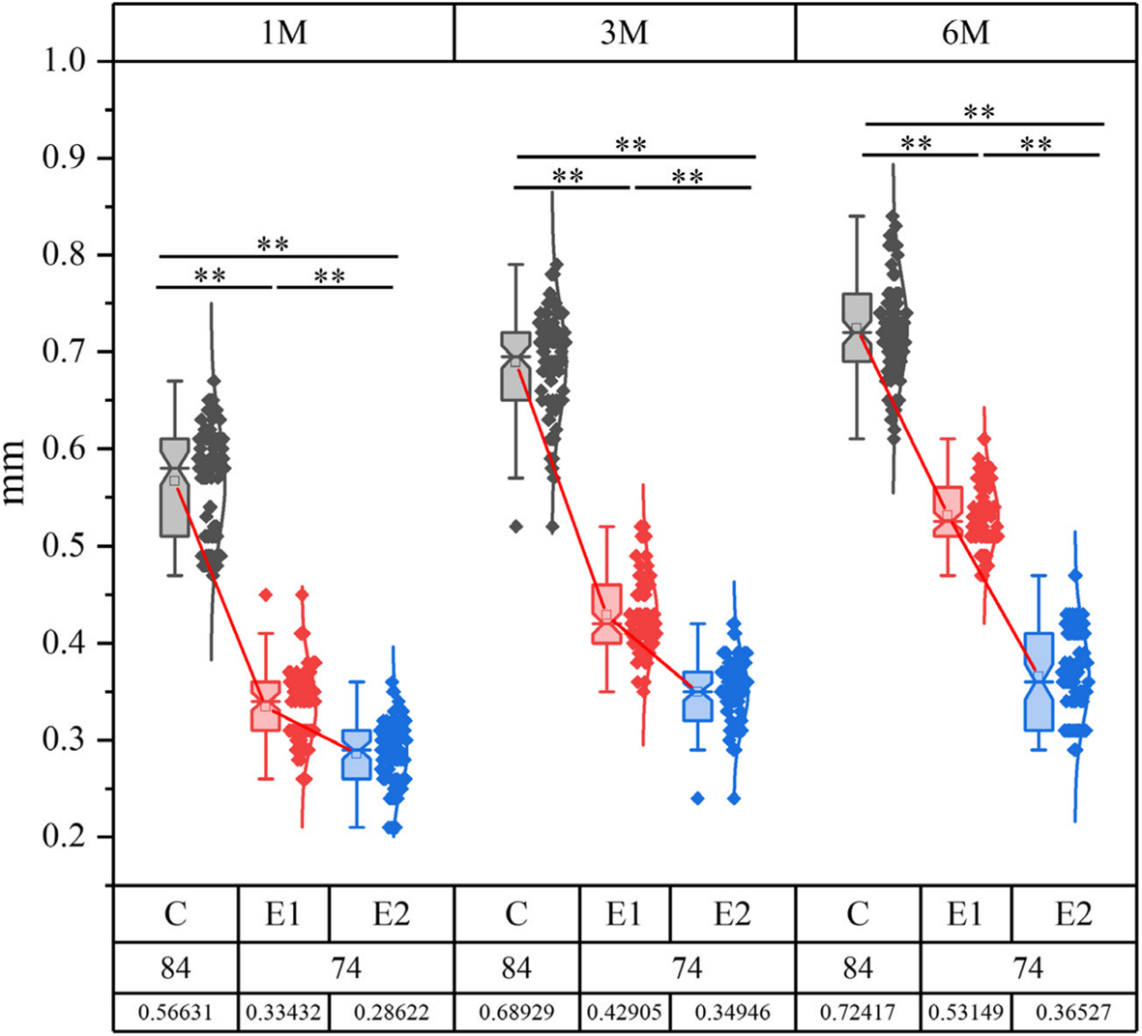
Comparison of the amount of gingival changes in the three groups.C:control group;E1:experimental group1;E2:experimental group2

The study analyzed the gingival changes in different groups at 1, 3, and 6 months after the procedure. The amount of gingival alteration was significantly less in patients using the 3D-printed platysma group and the dual-positioning guide group compared to the traditional platysma group (*P* < 0.01). Notably, the dual-positioning guide plate group had the least amount of gingival change. Refer to Fig. 8 for specific data.

### Post-operative satisfaction

**Fig. 9:**
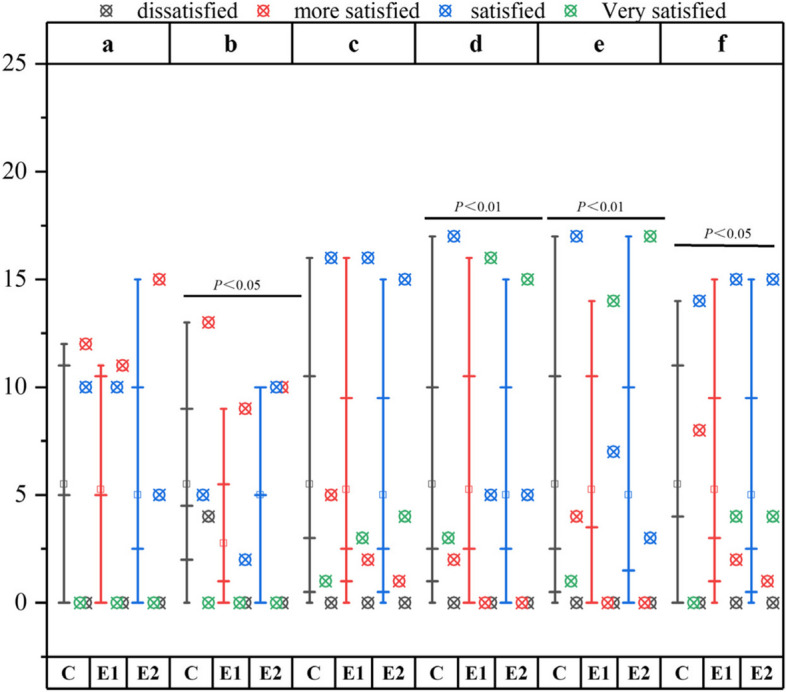
Comparison of Satisfaction of the Three Groups;**a**:costs;**b**:No. of visits;**c**:comfort of use;**d**:Restoration profile;**e**:Restoration Colour;**f**:overall satisfaction.C:control group;E1:experimental group1;E2:experimental group2

The study's statistical analysis revealed significant differences between patients in the experimental and control groups regarding the number of visits, restoration color, restoration shape, and overall satisfaction (*P* < 0.05, *P* < 0.01, *P* < 0.01, *P* < 0.05, respectively). However, there were no significant differences in terms of comfort and cost (*P* > 0.05) (refer to Fig. 1a for details). In comparison to the group using conventional compression membranes, both the group using 3D printed compression membranes and the group using dual positioning guides had a higher probability of achieving a very satisfactory or satisfactory appearance. Please refer to Fig. 9 for specific data.

### Secondary operations

A comparison of the incidence of secondary surgery using the traditional compression membrane group, the 3D printed compression membrane group, and the dual positioning guide group showed that the difference in the incidence of secondary surgery between the three groups was not statistically significant (*P* > 0.05). Specifically, the incidence of secondary surgery in the three groups was 9.1%, 4.8%, and 0%, respectively. Specific data can be found in Table 3.

**Table 3:** Three groups of secondary operations

**Groups**	**Occurrence of secondary surgery**	
	**Frequency**	**Percentage (%)**
Control group	2	9.1
Eperimental group 1	1	4.8
Eperimental group 2	0	0
C-E1(*P*-value)	0.578	
C-E2(*P*-value)	0.167	

## Discussion

Smiling is an important way to express emotions and plays a significant role in daily life. According to contemporary aesthetics, a beautiful smile is one where the right amount of teeth are exposed. The current stage of oral aesthetic restoration aims to combine natural beauty with the patient's beauty. In the clinic, restorers are increasingly pursuing the restoration of not only the patient's masticatory and speech functions, but also their natural, vivid, and real individual aesthetics. This approach aims to achieve a perfect unity of nature, science, and art [17].

When performing crown lengthening surgery to adjust the anterior aspect ratio for patients in the clinic, we typically do so before placing temporary restorations. This is done for a period of 6 weeks to 6 months to shape the gingiva [18]. In the past, periodontal crown lengthening surgery was performed without a guide plate, which often resulted in suboptimal personalized aesthetic restorative outcomes. With the advancement of medicine and increased awareness of aesthetics, some scholars have started to carve a diagnostic wax pattern on the patient's oral plaster cast. They then use the crown lengthening guide plate, which has significantly improved the patient's experience during the surgery and the aesthetic restorative results after the surgery, compared to crown lengthening surgery performed with bare hands [19].

During gingival contouring, traditional self-coagulating temporary restorations and composite resin temporary restorations are not recommended for anterior teeth due to their potential to irritate the gingiva during fabrication, difficulty in secondary contouring, and increased risk of gingival recession and secondary caries with prolonged use [20]. The development of digital and 3D printing technology has made it possible to achieve personalized aesthetic restoration of anterior teeth [21, 22]. Additionally, 3D printing technology is now widely used in the creation of complete dentures, removable partial dentures, and fixed and implant-supported prostheses [23–27]. Therefore, the use of digital dental technology to guide the oral diagnosis and treatment process is an inevitable trend in the development of dentistry.

In the field of aesthetics, crown lengthening is often performed using guides to determine the final position of the gingiva. Common guides include resin crown guides, diagnostic wax-up guides, and compression-molded guides. The project team investigated three types of crown lengthening guides a 3D printed rigid guide, a 3D printed soft guide, and a guide made by printing the virtual crown model designed by DSD and then using a compression film to create the guide. In clinical applications, it has been observed that the 3D-printed rigid crown extension guide may cause mucosal inversion and is prone to breakage when placed in the oral cavity. Additionally, if the guide is printed too thick, it may impede the field of vision and the crown extension surgery. The 3D-printed soft crown extension guide is not precise enough due to its edge, making it difficult to accurately guide during clinical crown extension surgery. Directly printing hard or soft crown extension guides is both cumbersome and costly. The design of software for directly printing hard or soft crown extension guides is both cumbersome and expensive. Therefore, neither of the two types of crown extension guides mentioned above are suitable for clinical use. Due to the limitations of the clinical application of the crown lengthening guides mentioned above, we studied the third method in the early stage. This involved printing out an aesthetically designed 3D model and then using the traditional compression film method to make crown lengthening guides. This method has been shown to have a better clinical effect [28]. This method avoids the error of determining the gingival margin with the plaster model. The film pressing method is more mature in clinic technology, and it has lower costs and time requirements. This results in shorter waiting times for patients. The film pressing machine has negative pressure, which allows it to fix the edge of the guide plate tightly inside the patient's mouth, ensuring the accuracy of the surgery as much as possible. Therefore, all crown extension guides used in experimental group 1 of this study were made using this method.

Experimental group 2 utilized 3D printing technology to produce precise dual-positioned crown extension guides based on the final ideal plan. These guides enabled the periodontist to accurately control the amount of hard and soft tissues to be removed, resulting in the restoration of the ideal gingival shape and position. The use of dual-positioning crown extension guides simplified the surgical procedure, improved treatment precision, increased predictability, and enhanced patient comfort [29, 30].

Based on our analysis, we have concluded that the number of follow-up visits, guide plate design, and production time for the 3D printing pressure film group and dual-positioning guide plate group are significantly lower than those for the traditional pressure film guide plate group. The digital guide plate is designed using digital means in the early stages, allowing the periodontist, restorative dentist, and technician to share the platform and communicate with the patient online. This enables the patient to achieve the desired restorative effect without the need for multiple follow-up visits to the hospital. In contrast, diagnostic wax-up requires significant time for carving. Patients do not need to visit the hospital multiple times to achieve the desired restorative effect. Additionally, the diagnostic wax-up process is time-consuming and requires carving, as well as adjustments based on the patient's personalized opinions during follow-up appointments. These factors can increase the number of follow-ups and treatment time for the patient. Furthermore, the group that used dual-positioning guides had a significantly shorter operative time compared to the group that used traditional compression membranes. This may be attributed to the precise edges of gingival and alveolar bone resection that were designed into the dual-positioning guides before surgery. The periodontist was able to accurately resect the gingiva and alveolar bone based on the edges of the guides during the surgical process. Surgical time is reduced, bleeding is minimized, and postoperative restorative outcomes are improved. Upon analysis of the aesthetic results of PES, it was found that the digital guide group outperformed the traditional restoration group in terms of marginal gingival level, proximal mesial gingival papilla, and distal mesial gingival papilla at 4 weeks and 3 months postoperatively. Additionally, at 6 months, the marginal gingival level of the digital group was superior to that of the traditional compression membrane group. This was due to the increased accuracy of the digitally designed and printed guides, which guided both groups in the alveolar bone resection process. The process of alveolar bone resection is guided by a digital guide plate generated through 3D printing. This greatly reduces errors and instability in the fixation of the bone surface that may occur when using traditional vacuum membrane surgical guides. In contrast, the traditional membrane guide group relies on the periodontist and the 'shu' aesthetic scale to complete the process of alveolar bone resection through freehand techniques. Good alveolar bone trimming can provide more accurate guidance for periodontal surgery. The bone plays an important role in guiding the recovery and growth of the gingiva [31–35]. Therefore, it has a positive effect on the recovery of the proximal and distal mesiodistal ging.

Regarding WES aesthetics, it was found that in the 1st and 3rd postoperative months, the 3D-printed laminate group and dual-positioning guide group were significantly superior to the conventional laminate group in terms of tooth shape, tooth contour, and volume, color (shade and purity), restoration surface texture, and transparency. In the 6th month, the digital group outperformed the conventional laminate group in terms of tooth shape, color, restoration surface texture, and transparency. The characteristics of the digital group were superior to those of the traditional restoration group. This was mainly because the 3D printed material was liquid photosensitive resin with different colors. The temporary restorations can be selected based on the color of the patient's abutment teeth to match the neighboring teeth. Additionally, the 3D-printed temporary restorations have smoother edges and a shape that is more suitable for the gingiva morphology after periodontal surgery. It can be printed as a single crown, making it convenient for patients to perform periodontal cleaning and maintenance. The single crown form also facilitates postoperative follow-up and temporary crown modification, enabling better gingival papilla induction [36]. Most temporary crowns made with resin at the chairside can only be used as continuous crowns. The color is relatively uniform, which may not meet the needs of patients with varying tooth colors. Additionally, the translucency and three-dimensional sense may be lacking [37]. Temporary crowns can be 3D printed as single crowns or continuous crowns. Continuous crowns have better retention than single crowns, and their gingival plasticity is better in the short term. However, after 3–6 years, the gingival plasticity of continuous crowns is still better than that of single crowns. Temporary restorations in the form of a single crown may facilitate periodontal maintenance for patients, but in cases where the clinical crown is shorter, they may be prone to falling off. Therefore, the decision to use a single crown or a crown form should be based on the actual situation of the patient's mouth. It is important to note that after 3–6 months, there is no difference in gingival plasticity effect between the two forms of temporary restorations. It is crucial to maintain objectivity and avoid subjective evaluations in clinical decision-making. If the clinical crown meets the retention requirements and the shaping time is sufficient, it is recommended to use a single-crown temporary restoration for gingival shaping. Our research shows that the shape, color, and surface texture of the temporary restorations decreased at 6 months compared to 3 months.

The timing of postoperative restorations is a significant concern for patients. Patients desire prompt restoration of their anterior teeth as they can impede aesthetics and daily communication. However, the timing of restorations is determined by the surgeon, and must strictly adhere to the degree of tissue healing to ensure the procedure's aesthetic efficacy [38]. The appropriate timing for postoperative restorations remains a topic of debate and is closely linked to the type of crown lengthening surgery, the amount of bone removed from the alveolar bone, and the thickness of the gingiva, as well as the postoperative outcome. The time frame varies from 6 weeks to 6 months, but it is generally believed that thick gingival types should be restored 2 months postoperatively and thin gingival types should be restored 6 months postoperatively [39, 40]. The study analyzed the gingival changes in different groups at 1, 3, and 6 months after surgery, using the baseline gingival position of 2 weeks postoperatively. The results showed that patients using 3D-printed platens and dual-positioning guides experienced significantly less gingival alteration compared to those in the traditional platen group. This difference was statistically significant. In the dual positioning guide group, the least amount of gingival change was observed, with the most pronounced change occurring at 4 weeks. There was less change observed between 3–6 months. Therefore, we recommend performing the final restoration at 3 months postoperatively for patients with medium-thick gingiva and when the crown is lengthened using a digital guide.

Based on the results of patient satisfaction, the experimental group exhibited significantly higher levels of satisfaction compared to the conventional group in terms of the number of visits, the color and shape of the restorations, as well as overall satisfaction. No significant differences were observed between the three groups in terms of comfort and cost. However, the probability of being very satisfied and satisfied was higher in the 3D printed membrane and dual positioning guide groups compared to the conventional membrane group.

There is a patient in the research process of this project was not included in the study group, the reason is that this project gingival contouring time follow-up observation to 6 months after surgery, the cycle is longer, the patient in the follow-up period of pregnancy twins, and then due to physical reasons for the fetal arrest of the abortion surgery, the patient's body hormone level changes and poor oral hygiene, to be again to review the gingival redness and swelling, probing bleeding obvious, and then the patient After the patient was treated with systemic periodontal therapy and hormonal drugs, the gingival condition was relieved, and ultimately a good aesthetic restoration effect was obtained. This case also further confirms that the maintenance of long-term periodontal health is a matter of concern and treatment. Oral aesthetics must be based on oral health, and the first criterion of oral aesthetics is whether oral health is maintained, followed by aesthetic efficacy. Poor oral hygiene can easily cause gingival inflammation, which can have a serious adverse effect on postoperative gingival healing and periodontal health and aesthetics.

This project emphasizes the importance of patient participation in the digital treatment process from both the patient and professional perspectives. It aims to minimize aesthetic differences between doctors and patients to achieve better treatment results. During the analysis and design stage, healthcare professionals design the appearance of the lips, teeth, and gums based on the patient's needs and aesthetic principles. In the temporary crown and gingiva shaping stage, patients can provide their input to guide the clinic in creating the final restoration. Patient approval is the ultimate measure of success for anterior smile aesthetics. The use of digital technology enables multiple doctors to share patient information, allowing synchronous communication of cases and formulation of plans across time and space on a login platform. This improves the treatment plan and saves consultation time. In summary, the digital treatment process enhances the overall outcome of pink-and-white aesthetics and improves patient engagement and satisfaction when compared to traditional anterior aesthetics for treating dual aesthetic deficiencies in teeth and periodontal soft tissues.

In summary, although 3D printing technology still has limitations in aesthetically restoring anterior teeth, it has made significant progress compared to traditional methods. It can assist doctors in developing detailed and personalized treatment plans, increase patient participation, and achieve satisfactory aesthetic results. This can help patients feel more confident and have a charming smile. If the patient's time and economic conditions allow, 3D printing technology is a better option for anterior teeth restoration and should be promoted for use in clinics.

## Data Availability

The datasets generated and/or analyzed during the current study are not publicly available due to privacy and ethical concerns but are available from the corresponding author on reasonable request.

## Abbreviations

PES: Pink Esthetic Score
WES: White Esthetic Score
DSD: Digital Smile Design
VAS: Visual analogue scale
ANOVA: Analysis of variance
